# Standardization of the Filovirus Plaque Assay for Use in Preclinical Studies

**DOI:** 10.3390/v4123511

**Published:** 2012-12-06

**Authors:** Amy C. Shurtleff, Julia E. Biggins, Ashley E. Keeney, Elizabeth E. Zumbrun, Holly A. Bloomfield, Ana Kuehne, Jennifer L. Audet, Kendra J. Alfson, Anthony Griffiths, Gene G. Olinger, Sina Bavari, for the Filovirus Animal Nonclinical Group (FANG) Assay Working Group

**Affiliations:** 1 Integrated Toxicology Division, United States Army Medical Research Institute of Infectious Diseases, 1425 Porter Street, Frederick, MD 21702, USA; E-Mail: amy.c.shurtleff.ctr@us.army.mil; 2 Virology Division, United States Army Medical Research Institute of Infectious Diseases, 1425 Porter Street, Frederick, MD 21702, USA; E-Mails: julia.biggins@us.army.mil (J.E.B.); ashley.keeney@us.army.mil (A.E.K.); ana.kuehne@us.army.mil (A.K.); jennifer.l.audet@us.army.mil (J.L.A.); gene.olinger@us.army.mil (G.G.O.); 3 Center for Aerobiological Studies, United States Army Medical Research Institute of Infectious Diseases, 1425 Porter Street, Frederick, MD 21702, USA; E-Mails: elizabeth.zumbrun@us.army.mil (E.E.Z.); holly.a.bloomfield@us.army.mil (H.A.B.); 4 Department of Virology and Immunology, Texas Biomedical Research Institute, San Antonio, TX 78227, USA; E-Mails: kalfson@txbiomed.org (K.J.A.); agriffiths@txbiomed.org (A.G.)

**Keywords:** plaque assay, filovirus, Ebola, ebolavirus, marburgvirus, Marburg virus, Vero

## Abstract

The filovirus plaque assay serves as the assay of choice to measure infectious virus in a cell culture, blood, or homogenized tissue sample. It has been in use for more than 30 years and is the generally accepted assay used to titrate virus in samples from animals treated with a potential antiviral therapeutic or vaccine. As these animal studies are required for the development of vaccines and therapeutics under the FDA Animal Rule, it is essential to have a standardized assay to compare their efficacies against the various filoviruses. Here, we present an evaluation of the conditions under which the filovirus plaque assay performs best for the Ebola virus Kikwit variant and the Angola variant of Marburg virus. The indicator cell type and source, inoculum volumes, length of incubation and general features of filovirus biology as visualized in the assay are addressed in terms of the impact on the sample viral titer calculations. These optimization studies have resulted in a plaque assay protocol which can be used for preclinical studies, and as a standardized protocol for use across institutions, to aid in data comparison. This protocol will be validated for use in GLP studies supporting advanced development of filovirus therapeutics and vaccines.

## 1. Introduction

Filoviruses are highly lethal zoonotic agents of viral hemorrhagic fevers that are of concern as emerging pathogens and potential biothreat agents. There are no available vaccines or therapeutics approved by the United States Food and Drug Administration (FDA) for the treatment of filovirus infections, so there is a great need for continued drug discovery efforts to protect against these lethal viruses. Recently, a committee comprised of multiple research institutes and government funding agencies, the Filovirus Animal Nonclinical Group (FANG), was assembled to enhance communication, coordinate efforts, and align product development, regulatory, and scientific resources regarding all aspects of filovirus medical countermeasures (MCM) development and FDA approval thereof. To streamline product development activities funded by government agencies, the FANG has suggested concentration of MCM development efforts against two ebolaviruses, Ebola virus (EBOV) variant Kikwit (isolate 9510621, referring to the sample’s Centers for Disease Control and Prevention (CDC) registration number), Sudan virus (SUDV) variant Gulu (isolate/CDC #200011676), and one Marburg virus (MARV) variant, Angola (isolate/CDC #200501379) [1]. Pivotal efficacy studies supporting development of a filovirus therapeutic under the Animal Rule must be conducted as Good Laboratory Practice-compliant studies (GLP, 21 CFR Part 58) to prove safety and efficacy of the compound, and ensure the integrity of the data collected. Here we have explored the viral plaque assay as the assay preferred by the FANG for filovirus titration, and which could play a role in approval of new anti‑filovirus vaccines and therapeutics under the FDA’s Animal Rule of Efficacy (21 CFR 314.600). Viral titer in a variety of biological samples from Animal Rule-driven studies can be quantified by performance of a FANG-standardized and validated viral plaque assay and included in the total data package for evaluation by the FDA [2]. We envision that the plaque assay can be used in these therapeutic or vaccine studies to quantify virus stocks shared among institutes and verify the challenge dose administered to the animals on these studies. Most importantly, the plaque assay can be used to measure the live viral load in the serum and tissues of treated *versus* untreated animals as a measure of therapeutic efficacy [3,4,5]. 

Accurate quantitation of virus particles in a sample is essential for any antiviral research program. Presence of virus, viral genetic material, or viral antigen can be measured in a variety of ways, such as fluorescence or electron microscopy, serology, polymerase chain reaction (PCR), or quantitative reverse transcription (RT) PCR methods. However, these methods do not assess the presence of replication-competent virus in a sample. Many types of viruses can be quantitated by TCID_50_ assay or plaque assay, in which one or more permissive cells in a monolayer can be infected by single plaque forming units of virus, each of which are sometimes equivalent to a single virion. Under the right conditions, infection will spread to neighboring cells to form a visible hole or “plaque” in the cell monolayer. The cell monolayer can then be stained with a dye such as crystal violet or neutral red, all plaques in the monolayer counted either by eye or under a microscope, and the titer of infectious virus in the original sample calculated. A full discussion of animal virus plaque assay theory, methods, and parameters is reviewed by Cooper [6]. 

Certain assay parameters will affect the ability of a virus plaque to form. The indicator cell line must be permissive for viral replication and plaque formation, and must achieve a confluent monolayer, which provides the platform for the assay. Historically, Vero cells, which are African green monkey (*Chlorocebus sabaeus*) kidney cells, have been widely used as an indicator cell line for hemorrhagic fever virus plaque formation since their discovery in 1962 [7]. Three lineages of Vero cells have been subcultured: Vero, Vero 76 and Vero E6 (C1008), each with different properties which make them useful for bunyavirus, arenavirus, flavivirus and filovirus plaque assays [7,8,9,10,11]. EBOV plaques were reported in early studies with fetal rhesus lung, SW-13 and Vero cells [8,9], while more recently published methods detail the use of Vero E6 for EBOV and MARV plaque assays [12,13,14]. In addition to cell line choice, the volume of virus inocula, optimal overlay matrix and concentration, and the staining product and its optimal concentration will affect the appearance and quality of plaques and whether they can be accurately counted [6,8,10,15]. The plaque assay has enabled progress in many important areas of filovirus research, such as epidemiology, diagnostics and vaccine and therapeutics discovery and development [3,4,14,16]. To date, an optimized and standardized plaque assay protocol does not exist among filovirus researchers at various institutions, a tool which would enable comparison of results among laboratories performing FANG-driven preclinical studies to develop filovirus MCMs.

The FANG has considered that optimization and validation of a single assay protocol are the first steps towards executing GLP-compliant studies for the FDA. Therefore, the work presented here was performed to evaluate the effect of various critical parameters on the filovirus plaque assay in order to optimize the assay. We designed experiments to investigate these parameters, including cell line, inoculum volume, overlay concentration, and their effect on visualization of the plaques. Our goal was to identify the optimal reagent or process for each of these parameters, which could ultimately enable validation of a standardized plaque assay protocol for use on FANG-driven GLP studies, as well as any other filovirus research initiative choosing to make use of this assay.

## 2. Results and Discussion

### 2.1. Choice of Cell Line, Timing for Cell Seeding, and Cell Passage

**Figure 1:**
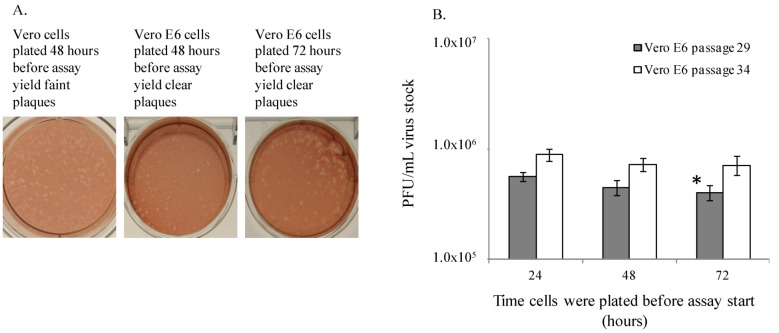
Vero E6 cells are suitable for quantitation of EBOV plaques. (**A**) Vero and Vero E6 cells produce EBOV plaques. (**B**) EBOV titers are similar in ATCC Vero E6 cells plated 24, 48 or 72 hours prior to plaque assay. This experiment was performed twice, and one representative graph is shown. Each bar represents an average of 5 replicates. The * indicates *p* = 0.006 between 24 and 72 hour samples for passage 29.

**Figure 2:**
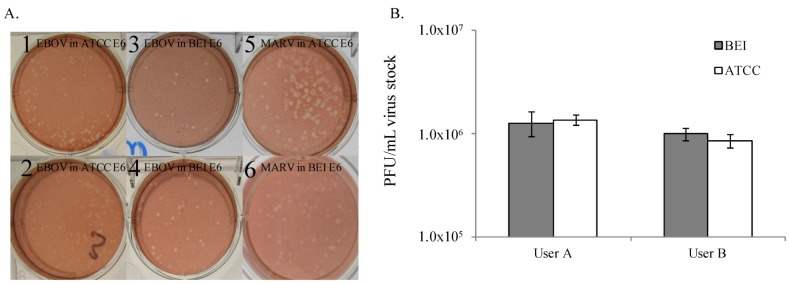
Filovirus plaques produced on Vero E6 cells from two sources are similar in appearance and titer. (**A**) EBOV plaques on ATCC Vero E6 cells plated at (1) 24 hours before assay, and (2) 72 hours before assay. EBOV plaques on BEI Vero E6 cells plated at (3) 24 hours before assay, and (4) 72 hours before assay. (5) MARV plaques on Vero cells from (5) ATCC and (6) BEI plated 24 hours before assay. (**B**) EBOV titers are similar in Vero E6 cells from ATCC and BEI when measured independently by two operators. This experiment was performed twice with up to 3 operators (data not shown), and one representative graph is shown. Each bar represents an average of 7 replicates.

Historically, Vero, Vero 76 and Vero E6 cells have been used to perform the filovirus plaque assay, but these cell lines have never been directly compared for performance. Therefore, the ability of Vero, Vero 76 and Vero E6 cells from American Type Culture Collection (ATCC, Manassas, VA, USA) to produce quality EBOV and MARV plaques was assessed (Figure 1 and Figure 2). Cells were seeded according to specific cell counts known through culture practices to produce 85%–100% confluent monolayers by the planned time of use (data not shown). Plaque assays were then performed on these cells 24, 48, or 72 hours post-seeding, using the same assay method each day. This experiment was performed twice, on different cell passages. ATCC Vero E6 cells at passage numbers 29 and 34 produced small, clear, easily quantifiable plaques on monolayers that had been in culture for 24, 48 or 72 hours prior to assay start. Moreover, the ATCC Vero E6 cells produced plaques that were superior to those produced on Vero 76 or Vero cells, at passages 39 and 44 or 129 and 134, respectively (Figure 1A). Vero 76 cells produced plaques that were visible and countable on cells plated 24 and 48 hours prior to assay start but plaques were not visible on cells plated at 72 hours before use. However, the plaques that formed on Vero 76 cells did not stain with as much contrast as the Vero E6 cells, thereby resulting in a light, unclear plaque against the stained monolayer background. The Vero cells generally produced unclear plaques that were hard to quantitate, and were the poorest quality of all three cell lines. Vero cell plaques were visible on cells plated at 24 hours prior to use, but not reliably quantifiable, with ill-defined borders and poor contrast. The only acceptable and countable plaques produced in Vero cells were in cells that had been in culture for 48 hours prior to assay, and no plaques were visible on cells plated 72 hours before use. These observations of plaque quality support the choice of Vero E6 cells over Vero or Vero 76 cells. 

In addition to plaque quality, it was also essential to examine the consistency of viral titers obtained from these three cell lines. The viral titers measured on ATCC Vero E6 cells plated 24, 48 or 72 hours before use ranged from 3.98 × 10^5^ PFU/mL to 5.56 × 10^5^ PFU/mL (cell passage 29), and from 6.99 × 10^5^ to 9.20 × 10^5^ (cell passage 34) (Figure 1B). The titers of EBOV stock material measured on ATCC Vero E6 cells maintain consistent titer values regardless of whether the cells were plated at 24, 48, or 72 hours prior to assay start. It should be noted that these titers are very similar despite the experimental design of performing plaque assays on three consecutive days with three vials of identical virus stock. The only statistically significant difference in viral titer was observed between passage 29 cells seeded 24 and 72 hours prior to use (*p* = 0.006, Figure 1B). These titration data indicate that similar results could be achieved using cells that had been plated for use up to 3 days before assay start. For performance of this protocol as a standardized assay, it may be advisable to prescribe a window of time post-seeding for use of the cells in the assay (e.g., 24–48 hours). 

Vero E6 cells from ATCC are available for purchase, but Vero E6 cells from BEI Resources, a NIAID-funded, ATCC-managed repository can be obtained free of charge in support of government funded projects, making them attractive for use in standardized assays. Vero E6 (BEI) originated from ATCC, and have been cultured at BEI Resources. To bridge the use of Vero E6 cells from ATCC to Vero E6 cells from BEI Resources, the two cell lines were tested twice in parallel assays, on cells plated 24 hours before use. No differences were observed in plaque quality or titer between Vero E6 cells from ATCC at passage 47 or BEI Resources at passage 31 (Figure 2), indicating that each cell line is equivalently reliable for use in the EBOV plaque assay. At the completion of EBOV experiments, Vero E6 cells from ATCC and BEI were evaluated in the MARV plaque assay. The assay worked equivalently well in both cell types, when tested at different times in separate assays. MARV titers ranged from 5.53 × 10^7^ PFU/mL to 6.67 × 10^7^ PFU/mL in ATCC Vero E6 cells and 7.6 × 10^7^ PFU/mL to 7.8 × 10^7^ PFU/mL in BEI Vero E6 cells in two different experiments with these cell types (data not shown). MARV plaque quality on BEI and ATCC Vero E6 cells appears equivalent (Figure 2A). In conclusion, Vero E6 cells from either ATCC or BEI Resources are acceptable for performance of the EBOV and MARV plaque assay. 

**Figure 3:**
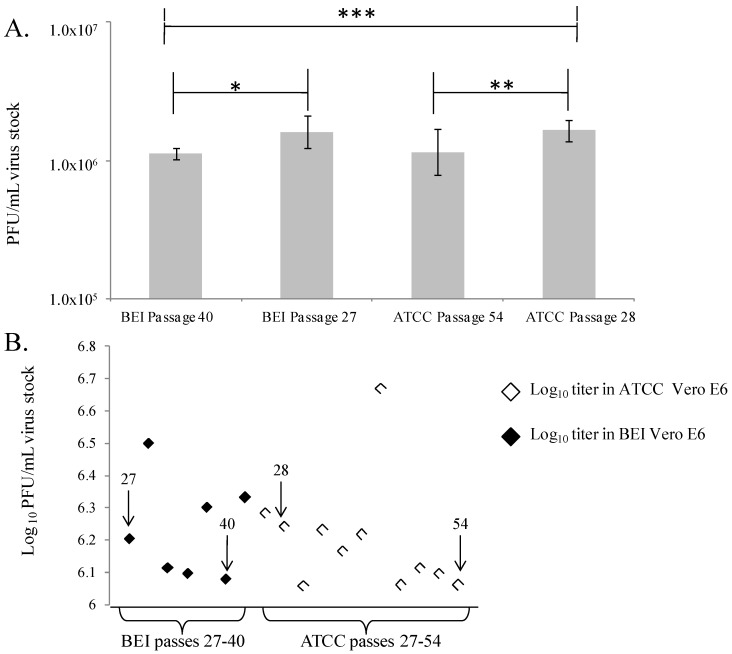
(**A**) EBOV titers in ATCC or BEI Vero E6 cells of various passage ages. This experiment was performed by three independent investigators, four replicates per cell type. The combined data are presented, where each bar represents 12 replicates. * indicates a significant difference in value between BEI passage 40 and passage 27 (*p* = 0.0003), ** a difference between ATCC passage 54 and 28 (*p* = 0.007), and *** a difference between BEI passage 40 and ATCC passage 28 (*p* = 0.000002). For these experiments, *p* value cutoff was ≤0.008). (**B**) Analysis of EBOV titer changes in cells of various passages. The arrows point out data from passages plotted in (A).

An additional parameter, performance of cells with increasing passage number, was important to assess because virus plaque formation and appearance may be affected by cell line age and time in culture. Therefore, head to head comparisons of virus titrations in Vero E6 cells from ATCC or BEI that had been in culture at USAMRIID for increasing lengths of time were performed three times in parallel with the same preparations of cells and with the same vial of seed stock virus (Figure 3A). Data from this experiment were pooled, and the average values were compared across all groups by Student’s t-test. Figure 3 demonstrates that there were incremental titer differences between cells of different passage ages. For the BEI Vero E6 cells, the virus titers measured were 1.6 × 10^6^ (coefficient of variation (CV) = 4%) and 1.1 × 10^6^ (CV = 12%), for the 27th and 40th passages, respectively (*p* < 0.008). For the ATCC Vero E6 cells, the virus titers measured were also 1.6 × 10^6^ (CV = 17%) and 1.1 × 10^6^ (CV = 8%), for the 28th and 54th passages, respectively (*p* < 0.008). These data may support a trend in reduction of titer in older cells, with a 0.5 log_10_ reduction in titer as cells remain in culture for weeks and are continually passaged. Additional experiments should be performed with larger numbers of replicate wells to further assign any importance to these results. Furthermore, a retrospective analysis of all plaque assay data collected in this program to optimize assay conditions does not reveal a trend of titer reduction as cells aged (Figure 3B). For this analysis, data from BEI cells at passages 27 to 40 (black diamonds), and from ATCC cells at passages 27 to 54 were plotted to determine if a trend across experiments existed that indicated any decrease in titer of the stock EBOV. In general, there did not appear to be a noticeable trend indicating any decrease in viral titer measured in the BEI Vero E6 cells, but there may be a slight trend in decrease of titer over time in the ATCC Vero E6 cells. Additional studies with high numbers of replicates should be performed to further test this preliminary observation. For standardization and validation studies, it will be critical to identify a window of acceptable cell passage number, and collect data using only cells within that range.

### 2.2. Cell Monolayer Confluency and Plaque Titer

**Figure 4:**
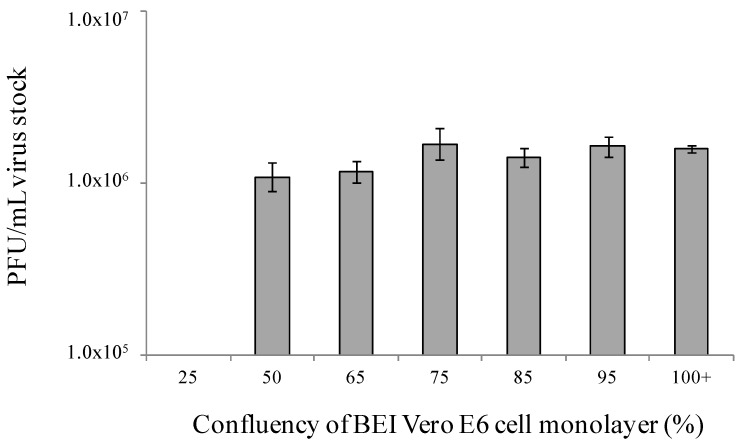
A wide range of monolayer confluencies returns a similar EBOV titer. This experiment was performed by three independent operators with similar results, and one representative graph is shown. Bars represent an average of 5 replicates.

The basis of any plaque assay is the formation of plaques in a cell monolayer. Monolayers with low cell confluency may yield lower or inconsistent virus titer measurements compared to highly confluent monolayers. In an initial experiment, titers were evaluated in BEI Vero E6 monolayers that ranged from 65% confluent to more than 100% confluent 24 hours after plating. Cells were counted and plated at numbers known to produce subconfluent, fully confluent, or heavily confluent, and therefore crowded monolayers. Monolayers were confirmed for percentage confluency by microscopic observation just prior to use. No statistically significant difference was observed in viral titers produced on these monolayers of varying confluency (*p* > 0.05). Titers across these monolayers ranged from 8 × 10^5^ PFU/mL to 1.2 × 10^6^ PFU/mL (data not shown). Given that similar titers were observed with monolayers exhibiting 65%–100% confluency, a second experiment was performed to include cells that were 25%, 50%, and 65%–100% confluent 24 hours after plating, to expand the lower end of the range of confluencies. Figure 4 demonstrates that monolayers plated to be 25% confluent did not produce countable plaques, and appeared mottled or patchy under neutral red stain by Day 8 of the assay. However, monolayers plated to be 50 to 100% confluent at the time of assay start produced similar titers with low variation, and no significant differences calculated (*p* > 0.05, and CVs ranging from 2% to 9%). These results strongly suggest that a monolayer with low confluency, such as 25%, is too low for EBOV plaque assay performance, but healthy monolayers at greater than 50 to 60% confluency will produce similar viral titers to 100% confluent monolayers. A narrower range of confluencies (85%–100%) will be chosen for assay standardization work and the validation protocol to simplify the experiments to verify cell confluency that must be included in the validation package. 

### 2.3. Assessment of Optimal Inoculum Volume

**Figure 5:**
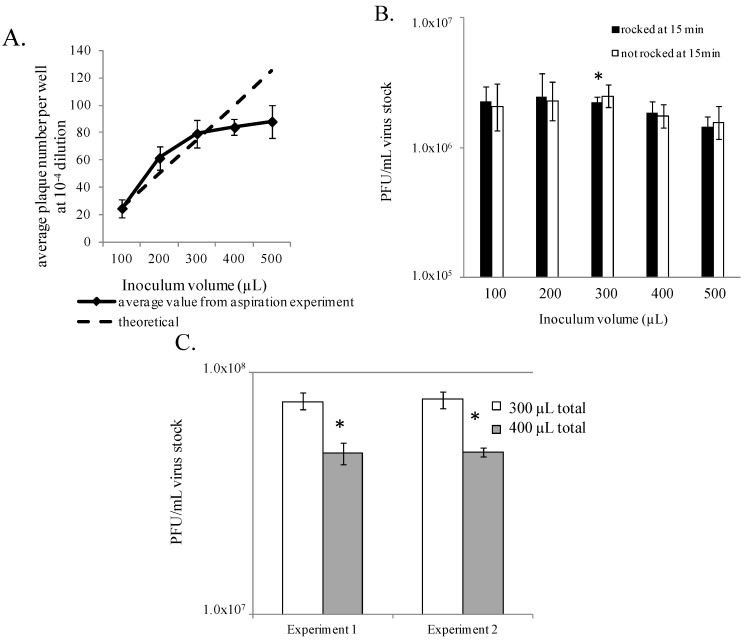
Evaluation of optimal inoculum volume. (**A**) EBOV titers may be reduced if the inoculum volume is too large. (**B**) 300 µL inoculum volume appears optimal for EBOV plaque formation. * indicates a significant difference in value between the 300 µL and the 500 µL conditions, where plates were rocked during incubation. Each bar represents six replicates (two experiments). (**C**) 300 µL inoculum volume appears optimal for MARV plaque formation. * indicates comparisons between 300 and 400 µL for Experiments 1 and 2, and across Experiments 1 and 2 resulted in p < 0.008. Each bar represents three replicates.

Generally, cells prepared in 6-well tissue culture plates (9.5 cm^2^ growth surface/well, such as CoStar, Corning, Inc., Corning, NY, USA) are cultured in 2–3 mL of cell culture medium per well, which must be removed prior to virus inoculation. Some investigators use a decanting method to remove the medium, where the entire plate is tipped over a receptacle inside the biosafety cabinet, using sterile technique. This method leaves behind a small amount of residual medium in the well. Careful measurement revealed that an average of 140 µL–170 µL of media remains in each of these wells (data not shown). Alternatively, some investigators aspirate the 2–3 mL medium from each well using a house vacuum system, vacuum pump, or pipettor. Removal by aspiration leaves no residual medium, but simply leaves the cell monolayer surface wet. This method requires attention to each well and can be done with sterile technique. Plaque assays performed using either method of medium removal demonstrated that both methods are acceptable as similar titers were observed in each assay (data not shown). The volume of inoculum in a well, as well as consistent distribution of the inoculum over the cell monolayer may affect the quantity and quality of plaques. The volume must be sufficient to prevent the cell monolayer from drying out during the incubation period, but also scant enough to force virus particles and the cell surfaces into close proximity to promote attachment and entry. To explore the optimal volume required for the plaque assay, various volumes (100 µL–500 µL) of diluted EBOV stock were added to cell monolayers from which the medium had been removed by aspiration. During the incubation period, one set of plates was rocked manually at 15–20 minute intervals to re‑distribute the inoculum liquid onto the monolayer, thereby avoiding uneven liquid pooling and development of dry areas in the well. A parallel set of plates was not rocked. The results indicated that 300 µL of inoculum volume was sufficient to maintain monolayer health. This volume also produced the least variability in plaque counts and calculated titer (CV = 4%). This volume generated an average viral titer of 2.2 × 10^6^ PFU/mL compared to an average titer of 1.4 × 10^6^ PFU/mL measured when plating 500 µL (*p* = 0.005), which is a 36% reduction in titer (Figure 5A). 

Given that all inoculum volumes yielded similar calculated titers, a theoretical line was plotted to determine the expected increase in plaque numbers appearing in the wells as the inoculum volume increases, if inoculum volume had no negative effect on plaque formation (Figure 5A). These theoretical values were based on a countable dilution from the 100 µL volume inocula where an average of 25 plaques per well was observed. The plaque counts from the EBOV assay followed the theoretical values until an inoculum of 400–500 µL was used. At this point, the plaque counts observed diverged from the counts determined with the lower inoculum volumes. This may suggest that the 400–500 µL inoculum volume is too large to provide accurate titers despite the similarity in titer to the 300 µL inoculum volume. The larger volumes may provide too much liquid space for efficient virus-cell interactions to occur and account for the wider variation in plaque counts observed at these volumes (CV 8%–13%). It was also observed that smaller inoculum volumes, such as 100 to 200 µL, led to monolayers that appeared to be damaged, presumably due to a drying effect. Damage to the monolayer confounded the plaque count, obscuring or destroying some of the plaques, and average titers calculated from these wells had greater variability (Figure 5A, CVs ranged from 12% to 19%). 

Rocking the plates during the incubation period did not appear to affect the test virus titer, but not rocking the plates did appear to promote damage to the monolayer, and led to greater variability in plaque counts especially in the wells that received 100 to 200 µL. Results from this EBOV experiment indicated that there was an optimal inoculum volume below which monolayer damage may have occurred. The least variable results came from use of an inoculum volume of 300 µL, with interim gentle rocking of the plates to prevent monolayer damage. This volume appeared to promote viral adhesion to the monolayer, and prevent damage due to drying effects. 

A similar experiment was performed evaluating the effect of 300 and 400 µL inoculum volumes on MARV plaque formation. Instead of aspiration methods, these plates were decanted to remove cell culture medium. Inoculum volumes of 100 µL and 200 µL were added to the residual volume in the decanted wells, therefore the total inoculums volume was 300 µL or 400 µL, respectively. All plates in this experiment were gently rocked manually at 15–20 minute intervals during incubation. The resulting virus titer from an inoculum volume of 300 µL was higher than that measured in wells inoculated with 400 µL (Figure 5C) (*p* < 0.008 for two separate experiments). The titers in the 400 µL inoculum sets, decreased by 39% in each experiment compared to the titers achieved by inoculating with 300 µL. These results indicated that the lower volume promoted a higher titer in the MARV plaque assay, a similar result to the observations made in the EBOV experiments above. Taken together, the results from these MARV and EBOV experiments indicate that 300 µL of fluid per well is optimal as the total virus inoculum volume on a cell monolayer, resulting in formation of plaques and robust titer data. Application of 300 µL total inoculum volume to the monolayer is recommended as a practice for the standardized assay.

The volume of available test sample that can be diluted and titrated by filovirus plaque assay can vary depending on its source, such as a small amount of animal serum or tissue homogenate, or a virus stock. We conducted a brief set of experiments to confirm that no difference was observed between the measured viral titers in a sample when initial volumes of EBOV sample as low as 15 µL or as high as 150 µL were used in a serial 10 fold dilution series (data not shown). Our tests supported the choice of any dilution volume procedure necessary to measure viral titers in small or large virus samples alike. No prescribed starting sample volume or dilution procedure is recommended for performance of the standardized assay or assay validation studies.

### 2.4. Optimization of Agarose Concentration

Achieving the correct primary overlay matrix for visualization of filovirus plaques is critical for accurate determination of the sample titer. Extremely low and extremely high concentrations of agarose in the primary overlay could have a negative effect on plaque quality and quantitation. The overlay is added after the inoculum has incubated on the monolayer for 1 hour at 37 °C. For all of these studies, there was no withdrawal of the inoculum or washing of the monolayer prior to the addition of overlay. To evaluate the optimal agarose overlay concentration for the EBOV and MARV plaque assays, the experiment was conducted using final agarose percentages from 0.20%–1.60% and 0.4%%–0.75%, respectively. Initially, several observations were noted during the handling of prepared agarose. Agarose preparations at final concentrations of 0.40%, 0.50%, and 0.75% were easy to handle during the overlay process, as they remained in a liquid state long enough to pipette, and then solidified soon after application to the wells. At the higher final concentrations of 1.00%, 1.25%, and 1.60%, the agarose began to solidify too quickly for pipetting during the overlay process, despite being melted in a similar fashion to the other agarose concentrations. At the lower final concentrations, 0.30% was easy to use but a prolonged time was required for this agarose to reach a solid state on the monolayer. The 0.2% final agarose concentration never fully solidified at any point during the plaque assay. Furthermore, there was an effect of agarose concentration on plaque size and appearance. The plaques were smaller and more difficult to distinguish at the higher agarose concentrations, and were hazy or unclear at lower concentrations (Table 1). At final concentrations of 0.50% and 0.75%, the plaques were clear and easy to read for both EBOV and MARV. The varying concentrations of agarose also appeared to have an effect on the Vero E6 monolayer (Table 1). Monolayer damage, such as tears and sloughing, was observed at extremely low and high concentrations of agarose in the overlay, but 0.5% consistently preserved the best monolayer health and appearance for EBOV and MARV plaque formation. 

The varied agarose concentrations did not have a great effect on the final titer data, except at the highest concentrations, where monolayer damage was severe enough to reduce plaque counts due to difficulty visualizing and quantifying plaques (data not shown). The least variability in titer across all three experimental iterations was measured under the 0.5% concentration, where the percent CV ranged from 1% to 4%. The titer measured at the 0.5% concentration was statistically different from that measured in the 1.25% and 1.60% concentrations of agarose in experiment 2, with about a 2-fold reduction in plaque count measured in these higher concentrations, where there was more monolayer damage due to agarose effects (data not shown). Similar observations were made in experiment 3, indicating that 0.5% agarose concentration was the overlay formula most conducive to handling, formation of clear countable plaques and resulted in the least monolayer damage.

**Table 1:** EBOV plaques appeared clear and easily countable when a final concentration of 0.5% agarose is applied to the wells. Data were collected in three experiments, where three to six replicate wells were observed for agarose concentration effect.

Final Agarose %	Plaque size and appearance	% Wells with monolayer damage (# damaged of total)
**0.20**	Majority of plaques were “hazy” or “fuzzy”, plaques were faint and difficult to distinguish from damaged areas of the monolayer	92% (33 of 36 wells)
**0.30**	Majority of plaques were “hazy” or “fuzzy”, plaques were faint and difficult to distinguish from damaged areas of the monolayer	83% (30 of 36 wells)
**0.40**	Clear, easy to read plaques with the exception of a few “hazy” or “fuzzy” plaques	15% (7 of 48 wells)
**0.50**	Clear, easy to read plaques	7% (12 of 175 wells)
**0.75**	Clear, easy to read plaques	13% (16 of 126 wells)
**1.00**	Plaques are clear but smaller and a little more difficult to read	27% (35 of 128 wells)
**1.25**	Plaques were clear but very small and more difficult to read	56% (72 of 128 wells)
**1.60**	Plaques were clear but very small and very difficult to read	100% (56 of 56 wells)

### 2.5. Evaluation of Neutral Red Products and Optimal Concentrations for Use

**Figure 6:**
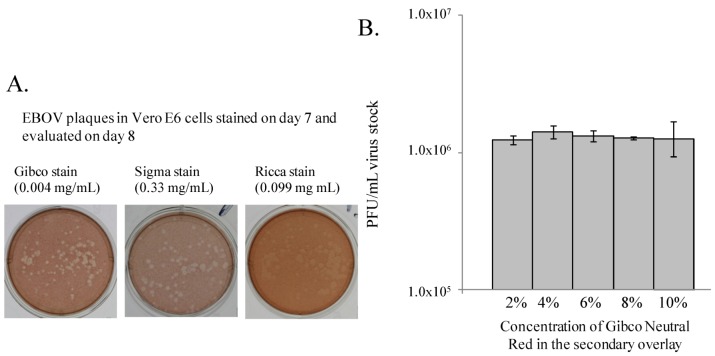
Gibco neutral red produced consistent results when tested at varying concentrations. (**A**) Three neutral red staining products provided different levels of staining quality. (**B**) Gibco neutral red produced consistent staining results when tested at varying concentrations. These experiments were performed twice with three replicates per experiment.

A sub-optimal staining product may yield faint staining and poor contrast of plaques against the surrounding monolayer, or even cause monolayer toxicity. Commonly used in plaque assays, neutral red is a vital dye which is taken up by live cells, and excluded by dead or metabolically inactive cells, such as infected cells remaining within a viral plaque [6]. Three commercial neutral red products were used in two separate experiments at concentrations suggested by their respective manufacturers to determine which product resulted in the most consistent, accurate, and easy to read plaques (Gibco (0.004 mg/mL), Sigma (0.33 mg/mL) and Ricca (0.099 mg/mL)). Staining the plates with these neutral red products did not yield any difference between the resulting plaque titers, which ranged from 2.1 × 10^6^ PFU/mL to 2.9 × 10^6^ PFU/mL for the first experiment, and 7.3 × 10^5^ PFU/mL to 8.4 × 10^5^ PFU/mL for the second experiment (data not shown). However, direct comparison of monolayers stained with the three products revealed that the Gibco neutral red product, which is specially prepared under contract for USAMRIID, provided the best contrast where the plaques could easily be distinguished from the surrounding stained, healthy monolayer (Figure 6A). The Sigma neutral red product yielded countable plaques, unlike the Ricca product. Our results support the use of a vital stain, such as the Gibco or Sigma neutral red, which produces sufficient contrast to yield plaques that can be easily seen and counted by the user. Evaluation of different types of neutral red was not performed in the MARV plaque assay. 

The Gibco and Sigma neutral red products were chosen for further evaluation to determine the optimal stain concentrations for adequate plaque development and minimal monolayer toxicity (Sigma product data not shown). For the Gibco stain, final concentrations (wt/vol) of 1% to 10% (0.001 mg/mL–0.010 mg/mL) stain in the secondary overlay were tested. Plaques from monolayers stained with 1% neutral red were too faint to count, and data were not collected. Similarly, the monolayers stained with only 2% neutral red produced plaques that were faint and difficult to count. The use of 8%, 10%, and to a lesser degree 6%, neutral red stain resulted in some toxicity to the monolayer. The monolayers stained with 4% neutral red produced the highest quality plaques, with no monolayer toxicity. There were no differences between the resulting viral titers obtained when the different percentages of Gibco and Sigma neutral red were tested (Figure 6B, and Sigma product data not shown). Considering the quality of the plaques and the titer data, the Gibco and Sigma neutral red products should be used at concentrations of 4% and 5%, respectively. The Gibco neutral red product produced optimal plaque counts at both 24 and 48 hours post-staining. Monolayers stained with 5% Sigma neutral red had optimal plaque counts at 24 hours post-staining, but when the plates were observed at 48 hours post-staining, some toxicity to the cell monolayer had developed, obscuring plaques. We concluded that staining monolayers with 4% Gibco neutral red provided the most clear and consistent plaque counts and the greatest flexibility for the choice of day on which to count plaques after stain was added.

### 2.6. Identification of Optimal Day to Stain and Count Plaques after Monolayer Infection

While the previously addressed assay parameters are vital for developing a standardized plaque assay, the optimal day(s) on which to stain the monolayer and then count the plaques may be the most important parameters to consider. To determine the appropriate day post-infection on which to stain the monolayers with neutral red, a plaque assay was performed to include staining on each of Days 5–8 post-infection with EBOV virus stock. The amount of time allowed for visualization of the plaques was also evaluated. For each day that the monolayers were stained, plaques were counted both 24 hours and 48 hours later. The titers measured across all days for which plaques could be counted in each plate set were similar (data not shown). Observations of plaque quality are provided in Table 2. Plaques in monolayers stained on Day 5 and counted either 24 or 48 hours post-staining were faint and difficult to read. Staining on Day 6 and counting plaques at 24 hours demonstrated small and difficult to count plaques, similar to plaques stained on Day 5 and read at 48 hours post-staining. However, plaques stained on Day 6 and counted at 48 h were still small in size but much easier to count. By Day 7, the plaques were large and easy to count at both 24 hours and 48 hours. Plaques stained on Day 8 were most easily read 24 hours post-staining. By 48 hours post-staining, the plaques were beginning to merge together and some toxicity to the cell monolayer was observed. Taken together, the results indicated that staining the monolayers on Day 7 yielded plaques with the best appearance and which were easiest to quantify. Staining on Day 7 also provided the greatest flexibility regarding the Day post-staining on which to count the plaques; plaques were read with similar ease at either 24 or 48 hours post-staining. 

**Table 2:** EBOV plaque observations based on various staining and counting days. These observations were made in two separate experiments with three replicate wells observed at each dilution in each experiment.

Day 5	Day 6	Day 7	Day 8	Day 9	Day 10
Stain	Very small, hard to count, many faint plaques	Small, hard to count, most plaques more visible than Day 6	N/A	N/A	N/A
	Stain	Small, hard to count, a few faint plaques	Small but easy to count, and no faint plaques.	N/A	N/A
		Stain	Easy to count, normal or average size	Larger, just starting to merge, still easy to count	N/A
			Stain	Larger and just starting to merge, still easy to count	Very large size, merging, slight toxicity in monolayer, more difficult to count

Subsequently, the optimal day to add stain in the MARV assay was evaluated. The results suggest that MARV infected cell monolayers stained on Day 7 and counted on Day 8 had plaques that were easiest to view overall. Differences in titer where cells were stained on Days 6, 7, or 8 and counted 24 or 48 hours later were minimal (data not shown). Taken together, these results indicate the best day to stain for both EBOV and MARV plaque assays is Day 7, with a window of two days during which plaques can be counted under neutral red. 

## 3. Experimental Section

*Virus.* A USAMRIID cell culture stock of Ebola virus (EBOV, isolate CDC#9510621) originated from an outbreak in Kikwit, Zaire in 1995, and was passaged twice at the Centers for Disease Control and Prevention (Atlanta, GA, USA), and once in Vero E6 cells at USAMRIID for a total of 3 passages. This stock is known to have a titer of 1.5 × 10^6^ PFU/mL using general plaque assay protocol methods at USAMRIID. A USAMRIID cell culture stock of an Angolan isolate of Marburg virus (MARV, isolate CDC#200501379), originated from an outbreak in Angola in 2005, and was passaged 4 times in Vero E6 cells. This stock is known to have a titer of 7.0 × 10^7^ PFU/mL using general plaque assay protocol methods at USAMRIID. 

*Cells*. Vero, Vero 76 or Vero E6 cells were purchased from American Type Culture Collection (ATCC, Manassas, VA). Catalog, lot and passage numbers at time of receipt at USAMRIID are as follows: Vero (Cat. No. CCL-81, lot 58484194, passage 123); Vero 76 (Cat. No. CRL-1587, lot 58078685, passage 33); Vero E6 (Cat. No. CRL-1586, 58027482, passage 23). Vero E6 cells were obtained from BEI Resources (Manassas, VA, USA). BEI Vero E6 cells are Cat NR-596, lot 3956812, passage 19. Passage numbers presented in the results section denote cumulative passage numbers upon receipt plus subsequent passages at USAMRIID. For example, ATCC Vero E6 cells were received at passage 23 and cultured and used up to passage 54 in the described work (Figure 1, Figure 2, Figure 3). All cells were cultured under well-documented conditions, where all medium, supplements and procedures for passaging and culturing cells were performed and recorded by qualified technicians. Cells were counted by use of either a Cedex HiRes Multi Sampler System (Roche, Indianapolis, IN, USA) or a hemocytometer. Cells were plated in 6-well tissue culture dishes (CoStar, Corning Inc. Corning, NY, USA) at appropriate concentrations and days prior to use, to achieve the desired percentage confluency, as determined by the experimental design. For most experiments, cells were used at 85%–100% confluency and seeded at 500,000 to 700,000 cells per well. This is a range of densities which will provide confluent cells for use at 24 hours (closer to 700,000 cells) to 48 hours post-seeding (closer to 500,000 cells). 

*Cell culture media, supplements and stains.* Reagents for culturing of cells: 1× MEM (Cat. No. 10010249, Cellgro, Manassas, VA, USA) in plates, supplemented with 5 to 10% FBS (vol/vol) (Cat. No. 16000-044, Gibco, Life Technologies, Grand Island, NY, USA), 5 mM L-glutamine (Cat. No. SH330034.01, Thermo Scientific HyClone, Logan, UT, USA). Reagents for plaque assay: 2× EBME (Cat. No. 05-5068EF, Gibco, Life Technologies, Grand Island, NY, USA), FBS (Cat. No. 16000-044, Gibco, Life Technologies, Grand Island, NY, USA), penicillin-streptomycin (Cat. No. 30-022-CI, Cellgro, Manassas, VA, USA), SeaKem ME Agarose (Cat. No. 50014, Lonza, Rockland, ME, USA), Neutral red from Gibco (Cat. No. 02-0066DG, Gibco, Life Technologies, Grand Island, NY, USA), Sigma (Cat. No. N2889, Sigma-Aldrich, St. Louis, MO, USA) and Ricca (Cat. No. 5271-4, Arlington, TX, USA). 

*Filovirus Plaque Assay.* Cells were plated in 6-well plates 24 to 72 hours prior to use in assay, and plates were carried into the BSL-4 lab. SeaKem ME Agarose (Lonza, Rockland, ME, USA) was prepared for overlays by mixing an appropriate amount of agarose with an appropriate amount of distilled water to achieve the desired concentration (*i.e.*, 1 g of agarose mixed with 100 mL of distilled water to achieve 1% agarose concentration). Agarose was autoclaved, allowed to solidify, and then melted by microwave at BSL-4 prior to use. Samples were diluted in 1× MEM, supplemented with 5% FBS (vol/vol), in a 10-fold fashion according to the experimental design. The culture medium in the 6-well plate was then removed by either swift decanting or aspiration by pipette and virus inocula of various concentrations were added to duplicate or replicate wells in volumes of 100 μL to 500 μL. Inoculum fluid was distributed by gentle rocking, manually. The plates were incubated at 37 °C for about 1 hour, with or without plate rocking every 15 to 20 minutes according to the experimental design. Without removal of the inoculum or washing the virus off of the monolayer in any way, a primary liquid overlay containing a 1:1 ratio of 2× EBME medium (supplemented with 10% FBS, 400 IU penicillin, 400 µg/mL streptomycin) equilibrated to 37–42 °C and 1% melted agarose equilibrated to 60°C degrees was overlaid in 2 ml aliquots onto the inoculated monolayer and allowed to solidify. Plates were incubated for approximately 7 days and stained with 2 mL of secondary overlay which consisted of 1:1 volume ratio of 2× EBME plus supplements: 1% agarose and the combined volumes were supplemented with 4% neutral red. Plaques were counted and recorded 24–48 hours post-staining. All work with filoviruses was carried out under maximum containment in the biosafety level 4 laboratories at USAMRIID.

*Plaque counting and titer calculation.* Plates were inverted on a light box and plaques were counted by hand with the aid of a marker, usually by one operator. For some experiments, plates were photographed to demonstrate plaque appearance and quality under various test conditions. Based on well size and plaque diameter, wells with less than 150 plaques and more than 10 plaques were used in the calculation of the titer [6]. If there were wells at two different serial dilutions with counts within this range, then the counts from wells with the more concentrated dilution were chosen for calculating titer. Counts from replicate wells were averaged, and that number was multiplied by the dilution factor of the inoculum which produced that count, and the volume of inoculum plated to calculate the plaque forming units (PFU) per ml of the original stock virus preparation. The calculation is: average value of plaques in replicate wells × mL of virus inoculum × dilution factor = titer in PFU/mL. 

*Variables under test.* Variables such as choice of Vero cell line, percentage confluency of cells in well monolayer, amount of time (in days) cells can be cultured in 6-well plates before use in assay, method of cell culture medium removal for initiation of assay, amount of virus inoculum volume, sample dilution procedures, requirement for interim plate rocking during incubation, percentage agarose in primary overlay, source of neutral red stain, concentration of stain(s) in secondary overlay, and optimal days upon which to stain and count plaques were all assessed as part of assay optimization. As experiments progressed over time, the “stability” of the cells, or effect of cell age (passage number) on plaque number and quality was evaluated in a specific experiment, and compared across all experiments, as appropriate. Specific details of the experimental design evaluating each of these variables are presented with the results for each variable.

*Statistical tests*. Raw data values were log-transformed for data analysis, and back transformed to present average viral titer values as mean ± standard deviation. Comparisons of sample groups were done assuming equal variance using two-tailed Student’s t-test in Microsoft Excel or GraphPad Prism software [17], with Bonferroni correction for multiple comparisons. Coefficients of variation (% CV) were calculated and are presented in the results where possible [18].

## 4. Conclusions

Generating consistent viral titer data in a plaque assay is contingent upon a number of parameters. These include monolayer cell type, cell culturing practices, cell confluency, virus sample inoculum volumes, final agarose concentrations in the primary and secondary overlays, staining product and concentration, and the optimal day for staining and counting plaques. Some of these parameters may be more critical than others. For instance, in early studies, ebolavirus plaques appeared under neutral red stain after 5–7 days in cultures which were incubated at 37 °C, but were not supplied with humidity or CO_2_ at the time, due to limitations in practices at BSL-4 [8]. The method of atmospheric control at that time was to wrap the plates in aluminum foil, and to leave them undisturbed in the incubator. Today, BSL-4 laboratories are fully equipped with humidity and CO_2_-controlled incubators, but ebolavirus plaques still tend to develop in 7 days, indicating culture conditions could be somewhat flexible. On the other hand, monolayer cell type is of critical importance. Vero, fetal rhesus lung, and human adenocarcinoma cells (SW-13) have all been reported to produce filovirus plaques with varying degrees of success, and a direct comparison of many lines indicated Vero cells were superior [8,9,19]. All of these parameters of varying importance must work in concert to produce clear countable plaques of a consistent size that result in reliable plaque titers. 

Similarly, parameters tested in this filovirus plaque assay optimization effort, were found to vary in their effect on virus titer outcome. For example, the results demonstrated that Vero E6 cells from either ATCC or BEI were better than Vero 76 or Vero cells from ATCC for the formation of filovirus plaques, which would suggest that it is important to assess the performance of possible cell types before choosing one type on which the assay will be based. While both BEI and its parent company, ATCC, provide Vero E6 cells which performed equivalently well, the BEI cell line has been chosen for use in FANG-standardized filovirus assays. BEI Resources has generated this stock of Vero E6 cells to be set aside as a special reagent resource dedicated to Department of Health and Human Services (DHHS) and Department of Defense (DoD) government grant and contract holders, and researchers performing work funded through these contracts will likely be required to procure and use these cells in filovirus plaque assays, especially for advanced development studies. Should groups running non-DHHS or non-DoD funded projects, but perhaps related projects, not be able to obtain these cells, our data support the use of ATCC Vero E6 cells as an acceptable substitute, if needed. 

The length of time cells were seeded prior to culture (24–72 hours) before assay start did not appear to affect virus titer (Figure 1B). There was only one statistically significant difference calculated between titers measured in cells plated at 24 *vs.* 72 hours before assay start (*p* = 0.006), but this observation was not repeatable. Therefore, it can be concluded that the small differences in titer across the days are not biologically significant in the assay, and the one significant difference measured may have been due to sample set size and low variation in the numeric values. Monolayer confluencies above 50% were important to virus titration, but surprisingly a heavily confluent monolayer was not required for consistent plaque formation and virus titration. Additionally, an inoculum volume of approximately 300 µL provided for optimal plaque titration for both EBOV and MARV, yet MARV may be more sensitive to this phenomenon, since a 100 µL increase in inoculum volume reduced titers by almost 40% (*p* < 0.008). Volumes lower than 300 µL resulted in monolayer damage, while higher volumes yielded decreased titers. Final agarose concentration, brand of neutral red product, and amount of stain did not affect plaque titer, but these variables can affect the quality of the monolayer in which plaques develop. If a monolayer were improperly overlaid or stained, the cells could be compromised and the plaque count confounded. Therefore, optimal overlay content and procedure are important parameters for collection of reproducible results. 

Other staining methods or overlay matrices were not evaluated in this filovirus plaque assay, although the semi-fluid Avicel® (FMC Biopolymer, Philadelphia, PA, USA) or methyl celluose overlays work well for influenza and hemorrhagic fever viruses [20,21] and are sometimes used at the institutes participating in these studies (USAMRIID, unpublished data [22]). Monolayers can also be fixed with 10% neutral buffered formalin and stained with crystal violet in ethanol, a practice which inactivates virus so completely that plaque assay plates can even be removed from containment laboratories, if desired. Photographs of the 6-well plates, rather than retaining the plates themselves, generally serve as sufficient records of plaque appearance, if required for future consideration.

Together with Department of Defense and the National Institutes of Allergy and Infectious Diseases funding agencies, USAMRIID and other BSL-4 laboratories in the United States, Canada and the United Kingdom that participate in the FANG have spearheaded research to develop filovirus medical countermeasures (MCMs) and the non-clinical assays to support this research. This research has resulted in the optimization and standardization of the FANG filovirus plaque assay protocol as a tool for use across multiple institutions, which will aid in the direct comparison of data generated at multiple worksites in support of FANG-directed studies. Government agencies intend to fund large intra-institutional advanced development contracts for filovirus therapeutics and vaccine development. Reproducibility of virus titer data internally and across institutions is paramount to the success of these programs. Additionally, the assay must be validated at each performing institution for use in GLP studies supporting therapeutic development under the FDA Animal Rule. A further intent of this study is to provide the filovirus research community with an assay protocol that could serve as a useful resource for performance of such a standardized assay. The studies presented here identified particular parameters that should be followed for performance of the standardized assay protocol, and helped to narrow the ranges of acceptable test variables when seeking to design SOPs and validation protocols for regulated studies (See Table 3). Furthermore, the results indicated that the assay performs well for both Ebola virus and Marburg virus. 

Previous experience at USAMRIID with Sudan virus (SUDV), which can behave slightly differently in culture from EBOV, leads the authors to hypothesize that this basic assay may function well for evaluation of plaques from other ebolaviruses or other filoviruses, but there may be some differences in the appropriate day to stain and count, or possibly other parameters. Continued collection of assay data and evaluation through formal validation protocols will be the best method to assess assay performance for all of the filoviruses of interest in advanced development studies.

**Table 3:** Standardized filovirus plaque assay suggested for use in FANG studies.

Assay Parameter	Recommendation for Standardized Assay based on Optimization Studies
Cell Type	Vero E6 cells from BEI Resources
Cell Passage Number	25 to at least 40, and higher passages may be acceptable upon further testing or institutional validation of the assay
Cell Plating Density	85%–100% confluent at time of usage, plates set up 24 to 48 hours prior using 500 to 700,000 cells per well
Inoculum volume	300 µL total inoculum volume
Final agarose concentration	0.5% SeaKem agarose
Neutral red	4% Gibco Neutral Red; 5% Sigma Neutral Red
Staining Day	7 days post-infection
Plaque Counting Day(s)	Gibco-24 and 48 hours post-staining
	Sigma-24 hours post-staining
